# Cognitive remediation therapy for anorexia nervosa as a rolling group intervention: Data from a longitudinal study in an eating disorders specialized inpatient unit

**DOI:** 10.1002/erv.2848

**Published:** 2021-06-12

**Authors:** Paolo Meneguzzo, Elena Tenconi, Patrizia Todisco, Angela Favaro

**Affiliations:** ^1^ Department of Neuroscience University of Padova Padova Italy; ^2^ Eating Disorders Unit Casa di Cura ‘Villa Margherita’ Arcugnano Vicenza Italy; ^3^ Padova Neuroscience Center University of Padova Padova Italy

**Keywords:** anorexia nervosa, cognitive remediation therapy, group therapy, inpatient treatment, rehabilitation

## Abstract

**Objective:**

Cognitive remediation therapy (CRT) has been proposed as an add‐on treatment approach that could increase the engagement in treatment of anorexia nervosa (AN) patients and reduce maintaining factors, but prior studies have evaluated CRT in individual and group settings, difficult protocols for rehabilitation settings. Our aim is to evaluate the CRT rolling protocol implementation in an inpatient specialised unit.

**Methods:**

A historical longitudinal controlled study was designed to include 31 AN patients for the CRT program, and 28 AN patients treated as usual. The CRT rolling group was implemented in a multidisciplinary inpatient rehabilitation ward with both adolescent and adult patients and an 8‐weeks protocol. To evaluate the treatment implementation effect, different self‐administered questionnaires were used.

**Results:**

The study found greater improvements of the CRT group in clinical symptomatology (*p* = 0.039), flexibility (*p* = 0.003), self‐confidence about the ability to change (*p* < 0.001), and less short‐term focus (*p* < 0.001), with no differences between restrictive and binge‐purging patients.

**Conclusion:**

This study demonstrates that CRT rolling group protocol is feasible in an inpatient treatment setting and may improve a rehabilitation program's outcome. Our results have shown how CRT can influence cognitive styles considered AN maintenance factors, positively affecting both restrictive and binge‐purge type.

## Key Points

- Cognitive remediation therapy (CRT) could be implemented in an inpatient setting with a rolling protocol
- CRT improves flexibility, drive to change, and therapy engagement
- No differences between restrictive or binge/purge subgroups
- Both adolescent and adult patients improved their cognitive styles

**Abbreviations**
AN: Anorexia Nervosa
CRT: Cognitive Remediation Therapy
TAU: Treatment as Usual

## 1 INTRODUCTION

Anorexia nervosa (AN) is a severe psychiatric disorder characterised by food intake restriction, reduction in body weight below what are considered healthy levels, and an overestimation of one's body size (American Psychiatric Association, 2013; Behrens et al., 2020; Solmi et al., 2018). Anorexia nervosa is also distinguished by a psychopathological core that includes depressive symptomatology and high rates of anxiety and interpersonal and emotional impairments (Meneguzzo et al., 2020; Monteleone et al., 2019; Solmi et al., 2019). The AN onset is usually during adolescence and a specific neuropsychological profile, characterised by specific impairments of executive functions that are partially modified by weight recovery (Tenconi et al., 2021) has been proposed. Indeed, this neuropsychological profile has a strong link to treatment outcome and is characterised by poor cognitive flexibility, impaired central coherence, and inefficient visuospatial processing (Brown et al., 2018; Fuglset, 2019; Harper et al., 2017; Reville et al., 2016; Tenconi et al., 2010).

A specific cognitive treatment called cognitive remediation therapy (CRT) has been proposed as an add‐on or pretreatment to specific target‐oriented treatments, such as cognitive‐behavioural therapy or family‐based treatment, in order to improve cognition and modify the patients' neuropsychological profiles (Dandil et al., 2020; Lock et al., 2018; Tchanturia, 2014; Tchanturia et al., 2017). CRT specifically aims to modify cognitive inflexibility and attention to detail, and it has offered promising findings in both adolescent and adult patients, as well as in both individual and group settings (Giombini et al., 2017; Roberts, 2018; Tchanturia et al., 2017; Timko et al., 2018; Van Noort et al., 2016). Moreover, CRT has been shown to facilitate the patients' engagement during treatment, with high levels of appreciation due to the ability to carry over skills beyond the clinical setting (Giombini et al., 2018). However, some authors have pointed out the high levels of heterogeneity in the tasks and measures used for the evaluation of CRT effects, suggesting the need for more studies to confirm the effectiveness of the treatment (van Passel et al., 2020). Group CRT has been showed to be effective in the improvement of specific neurocognitive abilities, and the preliminary data has showed a specific effect on the intrinsic motivation to implement changes into everyday life (Danner et al., 2015). However, further research is needed to evaluate the implementation of CRT in the real world in order to highlight, for example, differences between restrictive or binge‐purge subtypes (Dahlgren & Ro, 2014; Hagan et al., 2020; Tchanturia et al., 2013). Previous findings suggested the evaluation of different cognitive rehabilitation approaches due to different central coherence impairments between restrictive and binge‐purge AN patients, with a worse performance in restrictive patients (Van Autreve et al., 2013), as well as greater impairment severity in the attention/vigilance domain in the binge‐purge subtype (Tamiya et al., 2018). However, more evidence is needed because there is no agreement in the literature data about the differences between different subtypes and different body mass indices (BMIs, Keegan et al., 2021).

The literature data have showed more robust evidence with relation to the efficacy of CRT implementation in adults (Dahlgren & Ro, 2014), with only preliminary data in adolescents (Giombini et al., 2018), although the results seem to be promising. Recent reviews and metaanalyses of the literature have pointed out the predominant data about CRT implementation in adult patients, calling for more studies with adolescent samples, especially for the promising effects on set‐shifting and obsessive symptomatology (Hagan et al., 2020; Tchanturia et al., 2017). To our knowledge, there is only one study in the literature that included in the same CRT groups both adolescent and adult patients (Sproch et al., 2019), showing no difficulties in the integration of the two ages, as well as some effectiveness in the improvement of neuropsychological profiles of adults.

In the literature, CRT has been administrated with different protocols: in a one‐to‐one setting, or in closed groups without the possibility of adding participants (Craig, 2006; Gordon & Hibbard, 2005; Kazantzis et al., 2018; Tchanturia & Smith, 2015). Individual and closed‐group therapies are very expensive and, for both practical and economic reasons, are not the main approach in psychiatric agencies, mental hospitals, and health clinics and services (Miller & Mason, 2012). In rehabilitation wards, patients stay for a limited amount of time, and closed groups may not be available to them due to the role of the inclusion in closed groups. The short duration of the hospitalisation limits the availability of non‐rolling interventions availability for patients, with a possible reduction of the effectiveness of the treatment. If, however, CRT is shown to significantly impact motivation to change and cognitive flexibility in closed settings (Genders & Tchanturia, 2010; Hagan et al., 2020), it should be taken into serious consideration as part of AN rehabilitation programs as a reinforcement of their efficacy. Indeed, previous studies have suggested that inpatient CRT could be helpful for adult patients, showing less clear and consistent results in adolescents and calling for more studies in this field (Harrison et al., 2018; Sproch et al., 2019).

This study aims to examine the feasibility and outcome effects of the implementation of rolling CRT group treatment in a rehabilitation treatment program for adolescent and adult patients with AN, with CRT groups composed by combined aged patients. Secondarily, it tries to evaluate any differences in the effect of the CRT program among AN clinical subgroups, looking for specific differences between restrictive and binge‐purge behaviours.

## 2 METHODS

A quasiexperimental design was identified as an ethically acceptable research approach for the evaluation of the implementation of a CRT protocol in an inpatient treatment setting since the positive effects of CRT are already known in the literature data (Ambrosius, 2007; Grepmair et al., 2007; Hagan et al., 2020). A historical longitudinal controlled study was designed, and the control group recruitment was carried out before implementing the CRT program. The same measures and inclusion/exclusion criteria that were decided for the outcome evaluation were applied at the beginning and end of the inpatient treatment for both groups.

All patients had a diagnosis of AN according to the DSM‐5 criteria (American Psychiatric Association, 2013). Criteria for exclusion were: (a) having a severe medical comorbidity (e.g., epilepsy), (b) having a drug dependence, (c) having a history of neurological trauma, (d) having a specific neuropsychological difficulty (e.g., dyslexia) or a certified intellectual disability. The assessment of the inclusion and exclusion criteria was performed with a psychiatric interview before the admission to the rehabilitation unit. None of the patients refused to participate in the study. Data from patients receiving treatment as usual (TAU) were collected between September 2018 and June 2019, before implementing the CRT protocol, which started in June 2019. Both CRT and TAU patients were recruited consecutively, during the first week after their admission in the Unit.

### 2.1 Participants

Consecutive patients admitted to the specialised inpatient treatment of the Casa di Cura Villa Margherita in Arcugnano (Vicenza, Italy) were enrolled from September 2018 to March 2020. A total of 28 patients was included in the analysis as TAU group, and a total of 31 patients agreed to participate in the CRT group during the inpatient treatment. There was one male patient in the TAU group and one in the CRT group. See Table 1 for demographic details. In the TAU group, 14 out of 28 patients (50%) had a restrictive type diagnosis, and 15 patients (54%) were below 18 years of age and classified as adolescents. All patients were under treatment with at least one medication: 27/28 with SSRIs, 14/28 with benzodiazepines, and 12/28 with second‐generation antipsychotics. The CRT group was composed of 18 patients (58%) with AN restrictive type diagnosis, and 16 patients (52%) were adolescents. As regards medications, 28/31 with SSRIs, 15/31 with benzodiazepines, and 15/31 with second‐generation antipsychotics.

**TABLE 1:** Cognitive remediation therapy protocol for rolling group

Meeting	Cognitive target	Exercise
1	Multitasking	Drawing invisible circles and real infinity signs; word‐search and colouring in; flexibility homework
2	Bigger picture thinking	Complex figures task; main idea task; flexibility homework
3	Switching	Illusions; stroop tasks; flexibility homework
4	Summary	Occupations task; flexibility homework
5	Bigger picture thinking	Embedded words task; word search task; estimating task; flexibility homework
6	Switching	Up and down task; prioritising task; search and count task; flexibility homework
7	Bigger picture thinking	‘How to’ exercises; complex figures task; flexibility homework
8	Summary	Maps task; flexibility homework

*Note:* All the exercises referred to the cognitive remediation therapy (CRT) manuals available at http://www.katetchanturia.com/publications.

All patients provided written informed consent to collect and store their clinical data for research studies at the beginning of the inpatient treatment. The internal revision committee of the Casa di Cura Villa Margherita approved the implementation of the standard multidisciplinary treatment with this protocol. The study complies with the provisions of the Declaration of Helsinki, and it is an implementation of standard clinical practice.

### 2.2 Measures

All participants were evaluated for specific eating psychopathology at the beginning and the end of the inpatient treatment, as part of the standard service treatment outcome evaluation. A standardised set of questionnaires was used for the evaluation of the specific eating psychopathology and for the clinical impairment:

- Eating Disorder Examination Questionnaire (EDE‐Q): the EDE‐Q is a well‐established self‐report questionnaire to assess eating disorder psychopathology and behaviours (Fairburn & Beglin, 1994). It comprises 22 items, rated according to a seven‐point forced‐choice format (0–6). The questionnaire provides information about ED's crucial behavioural features (e.g., binge eating, vomiting, laxative misuse), with higher scores reflecting greater symptom severity or frequency. There are four subscales: restraint, eating, shape, and weight concern. Cronbach's *α* = 0.860.
- Clinical Impairment Assessment (CIA): the CIA is a 16‐item self‐report measure of functional impairment secondary to ED psychopathology (Bohn et al., 2008). Its items probe impairment in life domains typically affected by an ED, such as mood, self‐perception, and cognitive and interpersonal functioning. Respondents use a four‐point Likert scale, with responses ranging from ‘not at all’ to ‘a lot.’ Global scores range from 0 to 48, with higher scores representing more significant impairment. Cronbach's *α* = 0.855.

Moreover, all participants completed specific questionnaires about flexibility, obsessive behaviours, and motivation to change (see questionnaires below) to evaluate the effectiveness of the CRT treatment over the TAU treatment. The questionnaires were selected after a brief review of the existing literature about cognitive evaluation, and had already been administered to both the adults and adolescent samples. The participants provided feedback at the end of the CRT group.

- Coping Flexibility Scale (CFS): the CFS evaluates the ability to discontinue an ineffective coping strategy using two subscales (Kato, 2012): the evaluation coping subscale (e.g., ‘If I feel that I have failed to cope with stress, I change the way in which I deal with stress’) and the adaptive coping subscale (e.g., ‘When a stressful situation has not improved, I try to think of other ways to cope with it’). Each subscale includes five items that participants rated on a four‐point Likert scale ranging from 0 (‘not applicable’) to 3 (‘very applicable’); higher scores indicate higher levels of ability. Cronbach's *α* = 0.821.
- Obsessive‐Compulsive Inventory‐Revised (OCI‐R): the OCI‐R is an 18‐item self‐report inventory that assesses the frequency and associated distress of six symptoms domains: washing, checking/doubting, obsessing, neutralising, ordering, and hoarding (Foa et al., 2002). Each subscale score ranges from 0 to 12, with higher scores indicating higher rates of obsessive‐compulsive behaviour; the clinical cut‐off of the total score is considered to be 18. Cronbach's *α* = 0.887.
- Resistance to Change Scale (RCS): the RCS is a 17‐item self‐related scale designed to measure an individual's inclination to resist changes (Oreg, 2003). There are four different subscales (routine seeking, emotional reaction to imposed change, cognitive rigidity, and short‐term focus), and each item elicits the answer in a 6 Likert scale from 1 (‘strongly disagree’) to 6 (‘strongly agree’). Higher scores indicate higher cognitive resistance to change. Cronbach's *α* = 0.793.
- Detail and Flexibility Questionnaire (DFlex): the DFlex is a 24‐item self‐related scale designed to assess cognitive rigidity and attention to detail and is a well‐established tool for evaluating CRT treatment (Marchiol et al., 2020; Roberts et al., 2011). The clinical cut‐off for the cognitive rigidity subscale is 53 and above, and for the attention to detail subscale, it is 44. Each item is presented using a rating Likert scale from 1 (‘strongly disagree’) to 6 (‘strongly agree’). Higher scores indicate higher levels of psychopathology. Cronbach's *α* = 0.891.
- Cognitive Flexibility Inventory (CFI): the CFI is a 20‐item self‐reported inventory developed to measure the cognitive flexibility necessary to generate adaptive thinking using two different subscales: control and alternatives (Dennis & Vander Wal, 2010). The responses range from 1 (‘strongly disagree’) to 7 (‘strongly agree’), with higher scores indicative of greater cognitive flexibility. Cronbach's *α* = 0.788.
- Motivational Ruler (MR): the MR is a self‐reported tool used to assess the importance of and ability to change (W. R. Miller & Rollnick, 2002). It consists of three questions: (a) the importance of change (‘How important is it for you to change and recover?’), (b) the ability to change (‘How confident are you in your ability to change and recover?’), and (c) a realistic evaluation of the ability to change (‘How much can you realistically change and recover?’). Each question is rated on a 10‐point Likert scale (from 1 = ‘not at all’ to 10 = ‘very much’). Higher scores indicate higher motivation to change.
- Feedback CRT questionnaire (post‐final session): this questionnaire was administered only to patients who participated in the CRT groups. The questionnaire consisted of nine items to be rated on a five‐point Likert scale (from 0 = ‘not at all’ to 4 = ‘a lot’): questions on whether they found sessions enjoyable (three items), useful (one item), had learnt any new skills (four items), and their opinion about the advisability of the group (one item). Besides, three qualitative questions asked the patients what they think they learnt, what could be improved, and whether they have any other suggestions.

### 2.3 The treatment protocol

The treatment protocol of the ‘Casa di Cura Villa Margherita’ ward (e.g., treatment as usual, TAU) is based on a cognitive‐behavioural multidisciplinary approach, with individual weekly psychotherapy sessions, a weekly psychotherapy group session, nutritional counselling, nursing care, meal planning, family treatment, psychoeducational group therapy, and psychopharmacologic treatment as needed. Specific third‐wave approaches are already implemented (e.g., mindfulness and sensorimotor therapy) with specific individualised targets on the patients' psychopathology (see also Todisco et al., 2020). The rehabilitation protocol is set on 8 weeks, due to the limitations of the inpatient treatment of the Italian health system laws. The patients admitted to the ward range from 14 to 60 years of age and have at least one outpatient treatment failed in their personal history. The implementation of the CRT protocol was the only change in the patients' treatment during the inpatient rehabilitation.

### 2.4 The cognitive remediation therapy group

The CRT group was implemented in the inpatient multidisciplinary treatment rehabilitation program of the ‘Casa di Cura Villa Margherita’ (see the previous section for more information about the treatment protocol). The group sessions were all conducted by the same therapist (PM) and supervised by an expert therapist (ET). As suggested by previous literature (Tchanturia et al., 2016), all sessions included different elements: psychoeducation, practical exercises, reflection, discussion and planning of homework, and challenges that participants were asked to attempt outside of the group. The aim of the rolling group was the same as that of the closed group: to practice global and flexible thinking with the support of peer group members, to increase motivation to change, and interpersonal interactions (Genders & Tchanturia, 2010). Due to the inpatient ward organisation, the CRT was structured as a rolling group treatment, where new patients begin treatment as others end it. Each session started with the explanation of the goals of the CRT group done by one of the participants to the newly admitted with the support of the group leader. Then, the group proceeded with several exercises following the group protocol, with continuous discussions relating to the exercises and the generalisation of the exercise into the everyday experiences (see Table 1 for CRT protocol). Each group ended with a general brainstorming and with an optional discussion of the homework. Due to the presence of adolescent and adult patients in the groups, the exercises and discussions were not aged oriented, as suggested by protocols for younger patients (Maiden et al., 2016). No difficulties emerged regarding the administration of CRT to a combined aged group, neither from the patients nor from the therapist, confirming its feasibility as already reported in the literature (Sproch et al., 2019).

The duration of the protocol was about eight weeks, with one session of about one hour per week, which covered the entire hospitalisation duration. A mean of six participants attended each session and all the included participants covered all the sessions. All sessions explored alternative thinking but not performance outcomes. Specific sessions addressed multitasking, cognitive flexibility, and big‐picture thinking. Group protocol was based on the CRT manual for AN (http://www.katetchanturia.com/publications), as well as prior literature (Abbate‐Daga et al., 2012; Tchanturia, 2014; Timko et al., 2018).

### 2.5 Statistical analyses

The collected data were examined for normality. Demographic and psychopathology scores were then analysed with a paired *t*‐test for the within‐group analysis. Cohen's d for paired samples was used for the evaluation of the effect size. For the between‐groups analysis, outcome measures from the first and final sessions were analysed with general linear models (GLM) for paired measures to evaluate the impact of the CRT protocol on score changes. Partial eta‐squared (*η*_*p*_^2^) was used for the evaluation of effect sizes for all of the outcome measures, with *η*_*p*_^2^ = 0.01 indicating a small effect size, *η*_*p*_^2^ = 0.06 indicating a medium effect size, and *η*_*p*_^2^ = 0.14 indicating a large effect size (Cohen, 2013; Lakens, 2013). Cronbach's alpha was used for the evaluation of the reliability and validity of the questionnaires included. The alpha was set at *p* < 0.05 for all of the analyses. Data analysis was performed using IBM SPSS Statistics 25.0 software (SPSS, Chicago, IL, USA).

## 3 RESULTS

All of the 31 patients enrolled for the CRT group performed all the eight sessions of rolling protocol, with no dropout. There were no significant differences in the two groups' composition (*χ* ^2^ = 0.120, *p* = 0.728). See Table 2 for the demographic and clinical characteristics of all participants.

**TABLE 2:** Demographic characteristics at T0

	CRT (*n* = 31)	TAU (*n* = 28)	T	*p*
Age	20.55 (4.44) [16–30]	20.36 (4.63) [15–30]	0.162	0.872
BMI	15.46 (1.78) [12.01–17.50]	15.61 (1.24) [13.95–17.50]	−0.390	0.698
EDE‐Q	3.75 (0.70) [2.50–5.50]	3.84 (0.96) [2.00–5.50]	−0.403	0.689
CIA	33.74 (6.42) [20.00–42.00]	35.89 (5.51) [20.00–48.00]	−1.373	0.175

*Note:* Table reports mean, standard deviation, and minimum and maximum value.

Abbreviations: BMI: body mass index; EDE‐Q: eating disorder examination questionnaire; CIA: clinical impairment assessment; CRT: cognitive remediation therapy; TAU: treated as usual.

### 3.1 T0 evaluation

No significant differences were found at baseline between CRT and TAU groups for all the psychological questionnaires used.

### 3.2 Outcome measures: Longitudinal assessment within groups

For the CRT group, the within group analysis showed a significant increase of the BMI (T1: 17.39 ± 1.40; *t*(30) = −9.028, *p* < 0.001, *d* = 1.621), an improvement of the EDE‐Q (T1: 2.38 ± 1.00; *t*(30) = 6.646, *p* < 0.001, *d* = 1.194), and of the CIA (T1: 18.90 ± 5.64; *t*(30) = 19.398, *p* < 0.001, *d* = 3.484) scores.

For the TAU group, the within group analysis showed a significant increase of the BMI (T1: 17.02 ± 1.27; *t*(27) = −7.943, *p* < 0.001, *d* = 1.501), an improvement of the EDE‐Q (T1: 2.66 ± 0.96; *t*(27) = 3.840, *p* = 0.001, *d* = 0.726), and of the CIA (T1: 23.79 ± 5.80; *t*(27) = 11.334, *p* < 0.001, *d* = 2.142) scores.

See Table 3 for the other clinical variables evaluated.

**TABLE 3:** Within‐group comparisons of the longitudinal effects of cognitive remediation therapy (CRT) program and treatment as usual (TAU) program

	CRT				TAU			
	T0	T1	t (*p*)	D	T0	T1	t (*p*)	d
CFS total	12.16 (4.31)	14.19 (4.88)	**−2.965 (0.006)**	0.533	11.21 (3.84)	11.50 (3.06)	−0.548 (0.588)	0.104
Evaluation coping	7.26 (2.24)	7.87 (2.49)	−1.420 (0.166)	0.255	6.71 (2.12)	6.50 (1.97)	0.721 (0.477)	0.136
Adapting coping	4.90 (2.64)	6.32 (3.20)	**−3.127 (0.004)**	0.562	4.50 (2.71)	5.00 (1.98)	−1.342 (0.191)	0.254
OCI‐R total	26.84 (12.65)	21.61 (10.63)	**3.377 (0.002)**	0.607	25.86 (12.26)	24.89 (11.35)	**2.495 (0.019)**	0.472
RCS total	71.84 (9.29)	65.19 (9.73)	**5.193 (<0.001)**	0.933	72.75 (14.70)	71.50 (13.15)	**2.241 (0.033)**	0.424
Routine seeking	20.52 (3.84)	18.71 (4.50)	**2.861 (0.008)**	0.514	20.50 (5.02)	20.36 (4.47)	0.779 (0.443)	0.147
Emotional reaction	18.90 (2.61)	17.71 (3.185)	**2.237 (0.033)**	0.402	18.32 (4.16)	17.96 (4.10)	1.674 (0.106)	0.316
Short term focus	17.84 (3.60)	15.32 (3.58)	**5.153 (<0.001)**	0.926	18.04 (4.33)	17.82 (3.92)	1.000 (0.326)	0.189
Cognitive rigidity	14.58 (3.92)	13.45 (3.53)	1.990 (0.056)	0.357	15.89 (4.63)	15.36 (4.17)	**2.948 (0.007)**	0.557
Dfelx total	85.16 (14.99)	77.26 (12.60)	**4.209 (<0.001)**	0.756	82.68 (18.16)	80.82 (18.52)	**2.709 (0.012)**	0.512
Cognitive rigidity	48.87 (7.53)	44.13 (6.75)	**4.677 (<0.001)**	0.840	45.96 (10.10)	44.79 (10.09)	**2.530 (0.018)**	0.478
Attention detail	36.29 (8.76)	33.13 (7.24)	**2.880 (0.007)**	0.517	36.71 (9.90)	36.04 (10.01)	**2.585 (0.015)**	0.489
CFI total	76.90 (18.87)	85.97 (17.10)	**−5.928 (<0.001)**	1.065	82.64 (15.78)	84.18 (13.28)	−1.094 (0.284)	0.207
Alternatives	53.94 (12.52)	59.26 (11.42)	**−4.000 (<0.001)**	0.718	59.75 (11.46)	60.82 (10.66)	−0.720 (0.477)	0.136
Control	22.97 (8.75)	26.71 (8.02)	**−6.360 (<0.001)**	1.142	22.89 (9.04)	23.36 (7.10)	−0.676 (0.505)	0.128
MR1	8.53 (1.93)	8.27 (1.80)	0.744 (0.463)	0.134	8.85 (1.56)	8.96 (1.37)	−0.618 (0.542)	0.117
MR2	4.57 (2.33)	6.77 (1.77)	**−4.328 (<0.001)**	0.777	5.37 (2.33)	4.85 (2.11)	1.192 (0.244)	0.225
MR3	5.33 (2.19)	7.43 (1.50)	**−4.288 (<0.001)**	0.770	5.77 (2.12)	5.04 (1.56)	**2.642 (0.014)**	0.499

*Note:* Table reports means and standard deviation for T0 and T1. Significant results are reported in bold.

Abbreviations: CFS: coping flexibility scale; OCI‐R: obsessive‐compulsive inventory‐revised; RCS: resistance to change; DFlex: detail and flexibility questionnaire; CFI: cognitive flexibility inventory; MR: motivational ruler.

### 3.3 Outcome measures: Longitudinal assessment between groups

**FIGURE 1:**
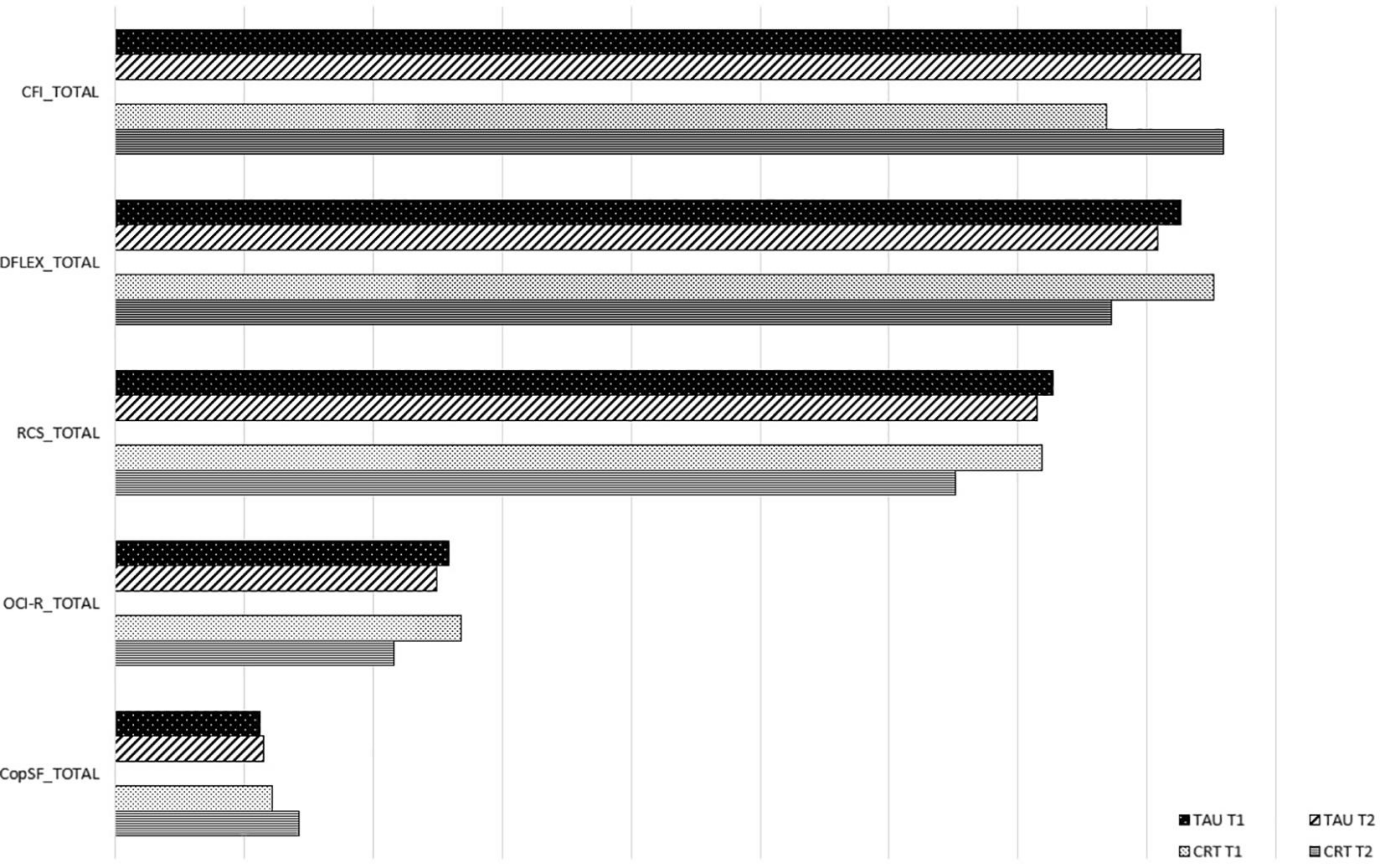
The figure showed the mean of the total scores of the self‐report questionnaire included in the study. Differences between T0 and T1, dividing cognitive remediation therapy (CRT) and treatment as usual (TAU) groups, are reported. The statistical analysis showed significant differences between couples for all the scales, with the CRT group that reporting greater effect sizes than the TAU groups in all the questionnaires

Both, CRT and TAU groups, at the end of the treatment (T1), showed similar BMI (*F*[57] = 3.522, *p* = 0.066) and similar EDE‐Q total score (*F*[57] = 0.301, *p* = 0.585). CIA scores, instead, showed a significant impact of the CRT treatment between the two groups (*F*[57] = 4.447, *p* = 0.039, *η*_*p*_^*2*^ = 0.072). All of the other measures were also tested using a GLM for repeated measures and showed a significant impact of the CRT treatment, with a large effect size for flexibility measures (as assessed by DFlex) and short‐term focus (see Table 3 for data). In Figure 1, we reported the global score of the self‐reported questionnaires included in the study, showing the differences reported in Tables 3 and 4.

**TABLE 4:** Between groups analysis of the effect of cognitive remediation therapy (CRT) program with a general linear models (GLM) analysis for repeated measures using treatment as a factor

	F	*p*	*η*_*p*_^2^
CFS total	**3.980**	**0.050**	**0.065**
Evaluation coping	2.390	0.128	0.040
Adapting coping	2.389	0.127	0.040
OCI‐R total	**6.507**	**0.013**	**0.102**
RCS total	**13.882**	**<0.001**	**0.196**
Routine seeking	**5.857**	**0.019**	**0.093**
Emotional reaction	1.960	0.167	0.033
Short term focus	**17.325**	**<0.001**	**0.233**
Cognitive rigidity	0.910	0.344	0.016
Dfelx total	**8.435**	**0.005**	**0.129**
Cognitive rigidity	**9.507**	**0.003**	**0.143**
Attention detail	**4.409**	**0.040**	**0.072**
CFI total	**12.900**	**0.001**	**0.185**
Alternatives	**4.567**	**0.037**	**0.074**
Control	**13.270**	**0.001**	**0.189**
MR1	0.829	0.367	0.015
MR2	**16.155**	**<0.001**	**0.227**
MR3	**23.901**	**<0.001**	**0.303**

*Note:* Significant results are reported in bold.

CFS: coping flexibility scale; OCI‐R: obsessive‐compulsive inventory‐revised; RCS: resistance to change; DFlex: detail and flexibility questionnaire; CFI: cognitive flexibility inventory; MR: motivational ruler.

### 3.4 Patient CRT feedback

**FIGURE 2:**
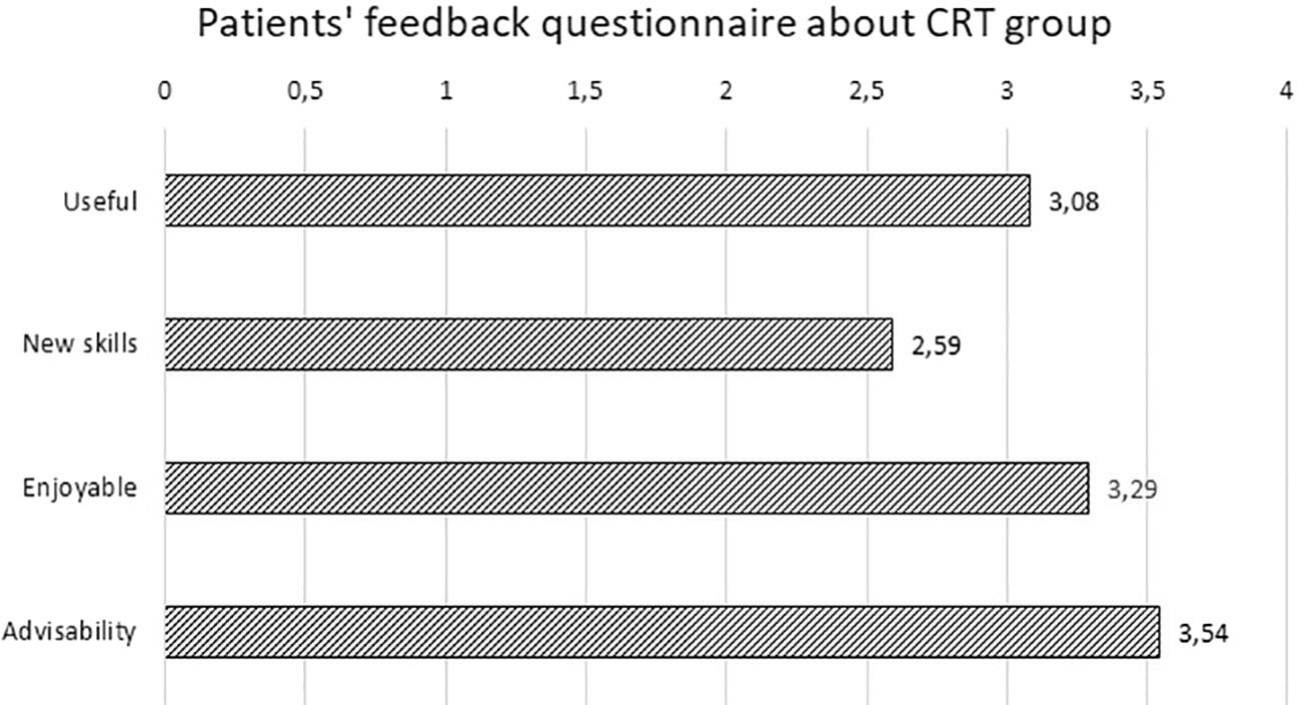
The figure shows mean scores on the cognitive remediation therapy (CRT) satisfaction questionnaire given after the last session. Patients were asked to quantify by a Likert scale (range 0‐4) how they found the group protocol enjoyable, useful, new skills enhancer, and advisable for others

None of the CRT participants gave a low score to the program. In Figure 2, the participant's opinion is summarised on a total of four points. On the open questionnaire, 28 participants out of 31 (90.3%) reported to have learnt new ways of approaching everyday activities, and 29 participants (93.5%) reported metacognitive improvement (i.e., they learnt more about their thinking style), as well as cognitive functioning and strategies. Only eight participants out of 31 (25.8%) reported that the treatment could be improved with more sessions, and 10 participants (32.3%) reported that more specific homework should be implemented to cover more everyday routines.

### 3.5 Adolescent versus adulthood

Different GLM analysis for repeated measures using adolescence/adulthood as factors between subjects reported no significant difference in any clinical or psychopathological scale considered.

### 3.6 Subclinical groups

Regarding diagnostic types, no significant differences were found between the AN restrictive and binge‐purge subgroups for the treatment effect on score changes, evaluated with several GLM analysis for repeated measurements.

## 4 DISCUSSION

This feasibility study was designed to evaluate the implementation of a rolling CRT group in an inpatient ward and assess the effect of CRT on outcome treatment measurements. Clinical and psychopathological evaluations were used as an instrument for evaluating the implementation of a rolling CRT program. To our knowledge, this is the first study of the feasibility of a CRT rolling group for AN. The treatment consisted of eight weekly sessions focussing on AN patients' main impaired neuropsychological domains: cognitive flexibility, central coherence, detailed thinking, and coping strategies such as problem solving, adjusting expectations, or seeking support.

Our data showed no significant effect on BMI or specific eating psychopathology between the CRT group and the TAU group, which is in line with previous studies with closed groups (Dahlgren & Ro, 2014). The CRT program does not focus on the eating concerns or dysfunctional eating behaviours that represent the core of AN, these are already treated using various psychological and nutritional approaches, but it moves beyond this core, targeting the cognitive disorder's maintaining factors (Watson & Bulik, 2013; Zhu et al., 2020). Indeed, our data showed improvements in the CRT participants as regards aspects linked to the psychosocial and cognitive domain. This improvement may bring patients to a greater awareness of the effects of AN on their lives, moving them to a more potent therapeutic alliance, that is, fundamental to AN recovery and for the modification of cognitive processing (Davies & Tchanturia, 2005; DeJong et al., 2012). This result is also confirmed by the comparison of the MR scores among the CRT and TAU groups. Indeed, CRT participants seemed to be more confident about their ability to change in their lives after treatment with a significant effect of the CRT training, perhaps because they experimented with some behavioural changes during the training protocol. Both CRT and TAU participants recognised the importance of change, indeed, the patients were voluntarily hospitalised in a psycho‐nutritional rehabilitation ward, but the CRT participants showed a robust increase in realistic confidence in their ability to change. This is in line with previous literature on CRT intervention and corroborates the utility of its implementation in AN treatment protocols (Denison‐Day et al., 2018; Tchanturia et al., 2016). Moreover, robust literature evidence suggests that motivation to change and its maintenance during the hospitalisation have a crucial role in the success of the treatment, corroborating the relevance of our results (Carter et al., 2012; Vall & Wade, 2015).

Specific aspects of the psychological domain, such as cognitive flexibility, central coherence, and short‐term focus, showed a significant improvement with the CRT rolling protocol. Indeed, in terms of efficacy of the implementation of CRT, medium to large effect sizes have been found with self‐reported psychological features measured in this study. These changes are the main effects of all of the CRT protocols available in the literature (Tchanturia et al., 2017), and our data corroborates this finding with a novel setting approach (i.e., rolling group), that is, also more adaptable to the real world, at least for the treatment services. An increase in cognitive flexibility could help patients accept changes in their habits and maintaining behaviours, with a possible long‐term effect (Dingemans et al., 2013), and our results are comparable with closed groups results (Pretorius et al., 2012). Besides, a reduction in short‐term attention to detail, together with an improvement in the ability to change and awareness, could help patients increase their investment in therapy to achieve recovery and life goals (Galimberti et al., 2013). Finally, improvements in the central coherence domain could help patients work on putative endophenotype aspects that require specific work, which could be a relapse factor (Dingemans et al., 2013; Tenconi et al., 2010). Indeed, the disengagement from the details and the development of more global thinking could help to accept useful changes in everyday life more than minimal eating‐focused changes, such as for dietary increments, which are characterised by cognitive rumination for minimal calories increase that usually bring to opposite effects, and help the recovery journey. No differences were found between adolescences and adults, nor between restrictive and binge‐purge patients, corroborating existing evidence of applicability of the CRT protocol in combined aged groups (Dahlgren & Ro, 2014; Hagan et al., 2020; Sproch et al., 2019; Zhu et al., 2020). Moreover, rolling groups with combined aged could allow more rehabilitation facilities to implement CRT protocol in their ED rehabilitation program, improving their outcomes.

Finally, in terms of the qualitative evaluation, rolling CRT groups may address its goals with excellent acceptability by patients, providing a link to real‐life behaviours that could help the rehabilitation process. All CRT studies have shown good feasibility and acceptance grades (Tchanturia et al., 2016). This more robust engagement could help the weak treatment commitment in psychological treatment for AN, increasing the share of goals and recovery using a self‐determination perspective (DeJong et al., 2012, Darcy et al., 2010), precisely because the patient experiences a different perspective on everyday habits. No differences raised by the rolling group protocol as regards acceptability or management difficulties, if compared to the previous literature of closed groups (Tchanturia et al., 2017). This improvement is an important result for the feasibility of the rolling CRT group because the literature showed how metacognition skills could play a role in the cognitive rehabilitation process of AN patients and this protocol is easily applicable in the rehabilitation facilities (Arbel et al., 2013; Sun et al., 2017).

## 5 STRENGTHS AND LIMITATIONS

This study has some notable strengths. This is the first study that evaluates a CRT rolling protocol for AN and its applicability in an inpatient setting. This approach could increase treatment accessibility without an increase in treatment cost. Besides, this study evaluates all significant psychological aspects of AN psychopathology, and it uses a control group with the same treatment and clinical characteristics of the CRT‐implemented group. Looking at the literature data, rolling group CRT seems to have a similar effect to that of the closed group, showing an increase in the cognitive abilities of the patients, and an improvement in the confidence in their ability to change disorder‐reinforced habits. However, it also has several limitations. The use of a historical control group with a quasiexperimental design (without randomisation) does not guarantee a total lack of performance bias and selection bias. The suitable matches between the BMI outcomes and the psychopathological outcomes may categorise our TAU group as a valid control for the evaluation of the protocol effects. Another possible limitation of the study is the lack of a follow‐up evaluation after discharge, but it should also be considered a proposal for future trials. Also, the lack of a neuropsychological assessment of patients does not allow us to evaluate the real effectiveness of the CRT implementation in modifying the cognitive performances of participants. However, this feasibility study focused on implementing a rolling group as an enhancer of standard multidisciplinary treatment for AN, and future studies might focus on cognitive neuropsychological profiles assessment.

## 6 CONCLUSIONS

This study replicates and extends previous evidence about the efficacy of CRT in AN treatment. Our findings showed that, after CRT intervention, the patients appear more motivated to change and their cognitive flexibility, central coherence abilities, and future‐term focus have improved. The feasibility of this rolling protocol and the positive feedback received supports the need for more studies on CRT as an open‐group treatment in AN and its implementation in inpatient treatment protocols also in combined adolescent and adult setting. Future studies should include people with different eating disorder diagnoses as suggested by previous literature and should consider implementing a specific module about emotions in the rolling group set. Moreover, randomised controlled trials are needed for more robust evidence on the efficacy of the CRT implementation in eating disorders treatment, possibly along with neuropsychological evaluation of the changes.

## CONFLICT OF INTEREST

On behalf of all authors, the corresponding author states that there is no conflict of interest.

## AUTHOR CONTRIBUTIONS

Dr. Meneguzzo had full access to all of the data in the study and takes responsibility for the data integrity and the accuracy of the data analysis. Concept and design: Clinical intervention was developed by Dr. Meneguzzo and Prof. Tenconi. Analysis or interpretation of data: all authors. Draught of the manuscript: c and Prof. Tenconi. Critical revision of the manuscript for important intellectual content: all authors. Statistical analysis: Dr. Meneguzzo. Administrative, technical, or material support: Dr. Todisco and Prof. Favaro.

## CONSENT STATEMENT

Written informed consent was obtained from all patients at the beginning of the inpatient treatment.

## Data Availability

The data that support the findings of this study are available from the corresponding author, upon reasonable request.
